# Supplementary material for: Multigenerational Social Housing and Group-Rearing Enhance Female Reproductive Success in Captive Rhesus Macaques (Macaca mulatta)
Source: Biology (Basel). 2022 Jun 27;11(7):970. doi: 10.3390/biology11070970 (PMC9311885; doi:10.3390/biology11070970)
Supplement: Supplementary file 1 [file biology-11-00970-s001.zip › File S1 - Age analyses.pdf]

File S1 – Age analyses

Note that there was a female age difference between peer (mean age = 8.6 ± 0.4, N = 129) and multi-generational groups (mean age = 6.3 ± 0.3, N = 229) (Wilcoxon rank rum test, W = 10277, *p*-value = 1.8e-06) (Figure S1.a). Also, females were significantly older in the peer and hand-reared group (R0: mean age = 13.8 ± 0.5 years, N = 35) compared with the group-reared females (R1: mean age = 5.4 ± 0.2 years, N = 120; R2: mean age = 4.1 ± 0.1 years, N = 74) (Kruskal-Wallis test X^2 = 98.6, *p*-value < 2.2e-16, peer N = 129, multigenerational N = 229; Benjamini & Hochberg post-hoc comparison of R0-R1 *p*-value < 2e-16, R0-R2 *p*-value < 2e-16, R1-R2 *p*-value = 5.2e-5) (Figure S1.b).

However, we found no correlation between age and birth rate, inter birth interval, offspring survival rate to first and third year of age (Figure S2).

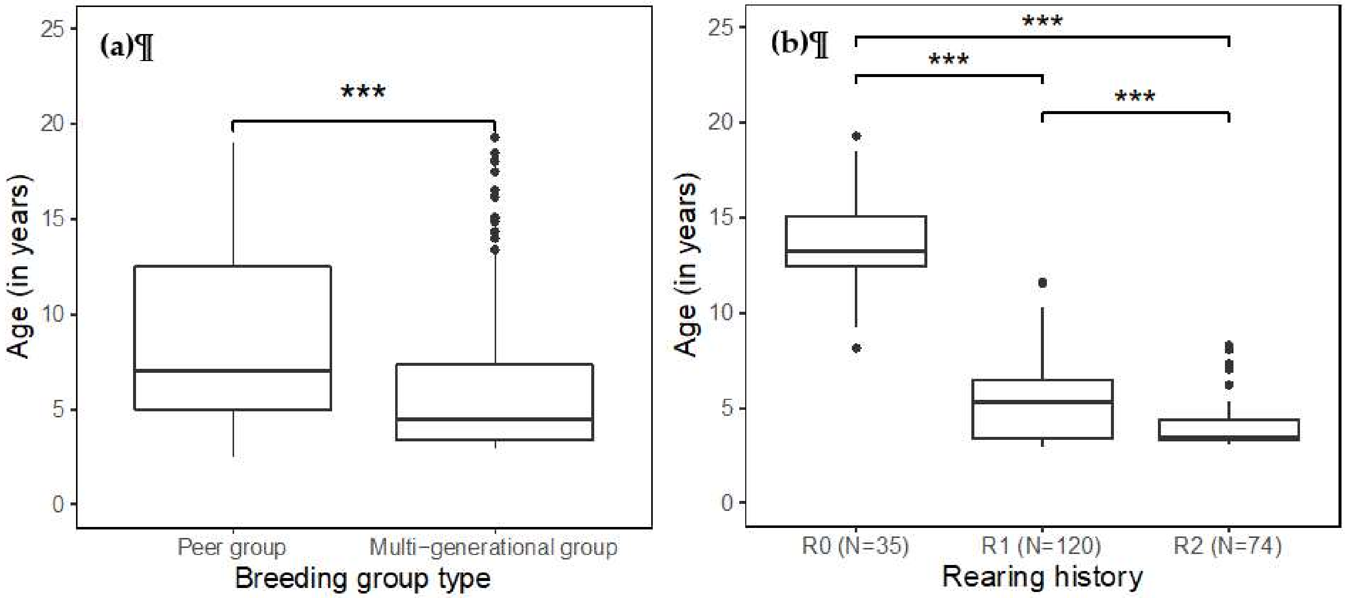

Figure S1. Age difference between **(a)** peer breeding group (N = 129) and multi-generational breeding group (N = 229) (Wilcoxon rank rum test W = 10277, *p*-value=1.8e-06). **(b)** Females were older in the hand and peer-reared group (i.e. R0) compared with the and group-reared groups (i.e. R1, R2) (Kruskal-Wallis test X^2 = 98.6, *p*-value < 2.2e-16, peer N = 129, multigenerational N = 229; Benjamini & Hochberg post-hoc comparison of F0-F1 *p*-value < 2e-16, R0-R2 *p*-value < 2e-16, R1-R2 *p*-value = 5.2e-5, R0: N = 35, R1 N = 120, R2 N = 74). *** indicates $p \leq 0.001$.

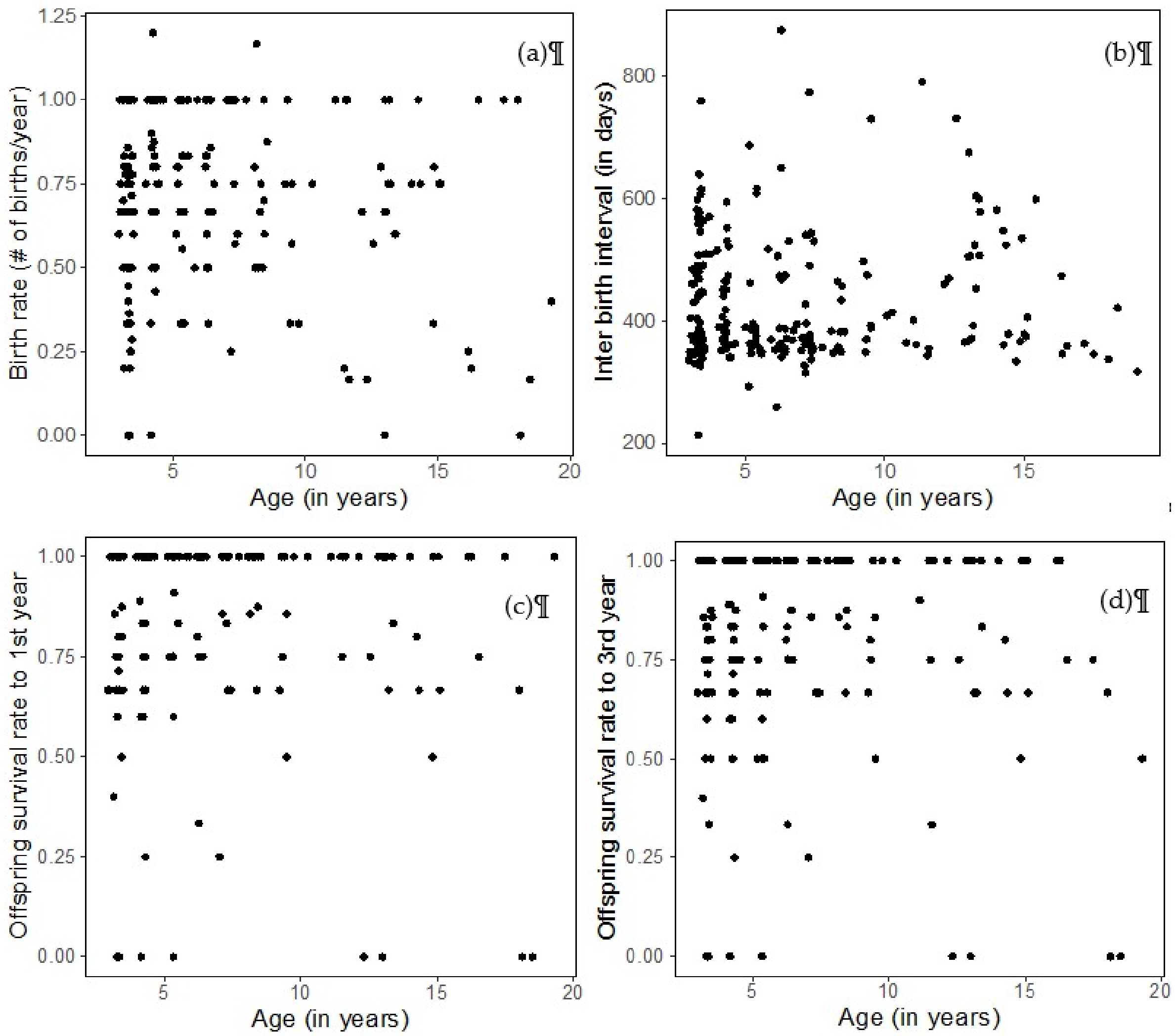

Figure S2. Correlation between female age and birth rate, inter birth interval (IBI), survival to first and third year of age. There was no correlation found between female age and **(a)** birth rate (Spearman's rank correlation: S = 1820103, p-value = 0.17, N = 229, **(b)** IBI (Spearman's rank correlation: S = 2056633, p-value = 0.68, N = 229), **(c)** survival to first (Spearman's rank correlation: S = 2096360, p-value = 0.48, N = 229), and **(d)** third year of age (Spearman's rank correlation: S = 2108887, p-value = 0.42, N = 229).
