# Supplementary material for: Multigenerational Social Housing and Group-Rearing Enhance Female Reproductive Success in Captive Rhesus Macaques (Macaca mulatta)
Source: Biology (Basel). 2022 Jun 27;11(7):970. doi: 10.3390/biology11070970 (PMC9311885; doi:10.3390/biology11070970)
Supplement: Supplementary file 1 [file biology-11-00970-s001.zip › File S2 - Statistical outcomes.pdf]

File S2 - Statistical outcomes

Table S1. The statistical outcomes of Wilcoxon rank sum test between breeding group type (i.e. peer vs. multi-generational) and female reproductive success (i.e. birth rate, inter birth rate, offspring survival to first and third year of age) in captive rhesus macaques.

	**Breeding group type**				W	*p*-value
	**Peer group**		Multi-generational group			
**Variable**	**Mean ± sd**	N (females)	Mean ± sd	N (females)		
Birth rate	0.47 ± 0.03	129	0.74 ± 0.02	229	7910	**1.93e-13**
Inter birth rate	448 ± 16	60	425 ± 7	169	5528.5	0.30
Offspring survival rate to 1st year of age	0.77 ± 0.34	107	0.90 ± 0.20	223	13909	**6.94e-03**
Offspring survival rate to 3rd year of age	0.74 ± 0.03	107	0.86 ± 0.01	223	13768	**0.01**
Conception	9.25 ± 0.53	60	8.50 ± 0.23	169	4611.5	0.30

Table S2. The statistical outcomes of Kruskal-Wallis test between rearing history (i.e. R0 = peer or hand-reared, R1 = group-reared with peer or hand-reared mother, R2 = group-reared) and female reproductive success (i.e. birth rate, inter birth rate, offspring survival to first and third year of age) in captive rhesus macaques.

	**Rearing history**						X^2	*p*-value
	**Peer or hand-reared (R0)**		Group-reared with peer or hand-reared mother (R1)		Group-reared (R2)			
**Variable**	**Mean ± sd**	**N**	Mean ± sd	N	Mean ± sd	N		
Birth rate	0.68 ± 0.05	35	0.77 ± 0.02	120	0.70 ± 0.03	74	2.97	0.23
Inter birth rate	426.9 ± 17.5	26	421.0 ± 8.4	99	428.2 ± 10.0	44	1.36	0.51
Offspring survival rate to 1st year of age	0.81 ± 0.05	33	0.91 ± 0.02	119	0.91 ± 0.02	71	6.8488	**0.03**
Offspring survival rate to 3rd year of age	0.78 ± 0.05	33	0.87 ± 0.02	119	0.88 ± 0.03	71	7.419	**0.02**

Table S3. The statistical outcomes of Kruskal-Wallis test with Benjamini & Hochberg post-hoc comparison of offspring survival rate to 1st and 3rd year of age between rearing history conditions (R0, R1, R2).

**Variable**		**R0**	**R1**
Offspring survival rate to 1st year of age	R1	0.055	-
	R2	0.035	0.370
Offspring survival rate to 3rd year of age	R1	0.072	-
	R2	0.025	0.186
